# Methane protects against polyethylene glycol-induced osmotic stress in maize by improving sugar and ascorbic acid metabolism

**DOI:** 10.1038/srep46185

**Published:** 2017-04-07

**Authors:** Bin Han, Xingliang Duan, Yu Wang, Kaikai Zhu, Jing Zhang, Ren Wang, Huali Hu, Fang Qi, Jincheng Pan, Yuanxin Yan, Wenbiao Shen

**Affiliations:** 1 College of Life Sciences, Laboratory Center of Life Sciences, Nanjing Agricultural University, Nanjing 210095, China; 2 Institute of Botany, Jiangsu Province and Chinese Academy of Sciences, Nanjing 210014, China; 3 Institute of Agricultural Products Processing, Jiangsu Academy of Agricultural Sciences, Nanjing 210014, China; 4 College of Agronomy, Nanjing Agricultural University, Nanjing 210095, China

## Abstract

Although aerobic methane (CH_4_) release from plants leads to an intense scientific and public controversy in the recent years, the potential functions of endogenous CH_4_ production in plants are still largely unknown. Here, we reported that polyethylene glycol (PEG)-induced osmotic stress significantly increased CH_4_ production and soluble sugar contents in maize (*Zea mays* L.) root tissues. These enhancements were more pronounced in the drought stress-tolerant cultivar Zhengdan 958 (ZD958) than in the drought stress-sensitive cultivar Zhongjiangyu No.1 (ZJY1). Exogenously applied 0.65 mM CH_4_ not only increased endogenous CH_4_ production, but also decreased the contents of thiobarbituric acid reactive substances. PEG-induced water deficit symptoms, such as decreased biomass and relative water contents in both root and shoot tissues, were also alleviated. These beneficial responses paralleled the increases in the contents of soluble sugar and the reduced ascorbic acid (AsA), and the ratio of AsA/dehydroascorbate (DHA). Further comparison of transcript profiles of some key enzymes in sugar and AsA metabolism suggested that CH_4_ might participate in sugar signaling, which in turn increased AsA production and recycling. Together, these results suggested that CH_4_ might function as a gaseous molecule that enhances osmotic stress tolerance in maize by modulating sugar and AsA metabolism.

Methane (CH_4_) is the most abundant reduced organic compound in the atmosphere, and also the second important greenhouse gas after carbon dioxide^1^,^2^. It was previously considered as a degradation product of organic substance by microbes under anoxic conditions^3^. Keppler *et al*.^4^ further reported a surprising discovery that terrestrial plants can produce CH_4_ under aerobic conditions. Although much controversy and debate followed this original work, a number of researchers have attempted to provide alternate explanations of the aerobic CH_4_ formation from plants using different scales of measurement^5^.

In fact, the non-microbial CH_4_ production from plants constitutes a significant fraction of the global CH_4_ sources^2^,^6^. A comprehensive understanding of CH_4_ in plants is that the living plants and fungi can not only emit CH_4_ to the atmosphere^7^, but also produce CH_4_ in plants *in vivo*^8^. Interestingly, this phenomenon was observed in the researches of nitric oxide^9^, carbon monoxide^10^, as well as hydrogen gas^11^. In some plant species, several chemical compounds, such as lignin, cellulose, leaf surface wax, ascorbic acid (AsA), the methyl groups of sulphur-containing amino acid methionine (Met), were suggested to be the potential precursors of CH_4_^2^,^5^,^12^,^13^. Based on the original work, it was further reported that the aerobic CH_4_ formation is significantly increased by ultraviolet (UV) radiation^14^, heat^15^, water stress^16^, bacterial pathogen^14^, and physical injury^17^. However, the potential function of endogenous CH_4_ for plant responses to various stresses is unclear.

Drought is the most pervasive limitation to the achievement of potential yield in maize, and triggers various interacting events including increased abscisic acid (ABA) and reactive oxygen species (ROS) levels^18^. This decreased availability of water is quantified as a decrease in water potential. During water deficit stress, osmotic stress sensing and signaling are pivotal to plant water status and lead to rapid changes in gene expression^19^. Normally, polyethylene glycol (PEG)-6000 is considered as the best solute for mimicking a water deficient stress that is reflective of the type of stress imposed by a drying soil^20^. Limited water availability caused by osmotic stress and drought, could provoke a shift in the balance between ROS production and their elimination in plants^21^. In fact, osmotic stress-induced ROS overproduction and corresponding oxidative reactions could result in the increases in lipid peroxidation and even the loss of biomass. For example, ROS overproduction is harmful to nucleic acids, proteins, lipids, and sugar if accumulated over a certain threshold in plants^22^,^23^. In order to avoid the overproduction of ROS caused by osmotic stress, plants have evolved specific antioxidant systems to ensure a control of the cellular redox state^24^, and these include the non-enzymatic components (ascorbic acid, AsA; glutathione, GSH; etc), and the antioxidant enzymes, such as superoxide dismutase (SOD), catalase (CAT), ascorbate peroxidase (APX), and guaiacol peroxidase (POD), etc^25^. Induced sugar metabolism is also observed when plants are subjected to short periods of oxidative or osmotic stress, suggesting that soluble sugars may function as osmoprotectants during stress^26^. Actually, sugars are being recognized as important regulatory molecules with both signaling and putative ROS scavenging functions in plants^27^. Since both CH_4_ and sugars (in particular) are involved in carbon metabolism, the relationship between CH_4_ and sugar metabolism under osmotic stress still remains to be elucidated.

To explore the potential role of CH_4_ in PEG-induced osmotic stress and corresponding mechanism(s), two maize cultivars (Zhengdan 958 and Zhongjiangyu No.1) differing in osmotic stress tolerance were compared in this study. Time course analysis revealed that CH_4_ production was induced by PEG-6000 treatment in maize root tissues, especially in osmotic stress-tolerant cultivar (Zhengdan 958; ZD958). Comparatively, more toxic responses were observed in osmotic stress-sensitive cultivar (Zhongjiangyu No.1; ZJY1). Subsequent work showed that CH_4_ pretreatment, not only mimicked the responses of PEG-6000 in the induction of endogenous CH_4_ production, but also alleviated PEG-induced osmotic stress by modulating sugar and AsA metabolism in maize seedlings. Meanwhile, the suppression of ROS production was observed. Our results therefore point to a beneficial role of CH_4_ in plant tolerance against osmotic stress.

## Results

### Phenotypic comparison of two maize cultivars under PEG treatment

**Figure 1:**
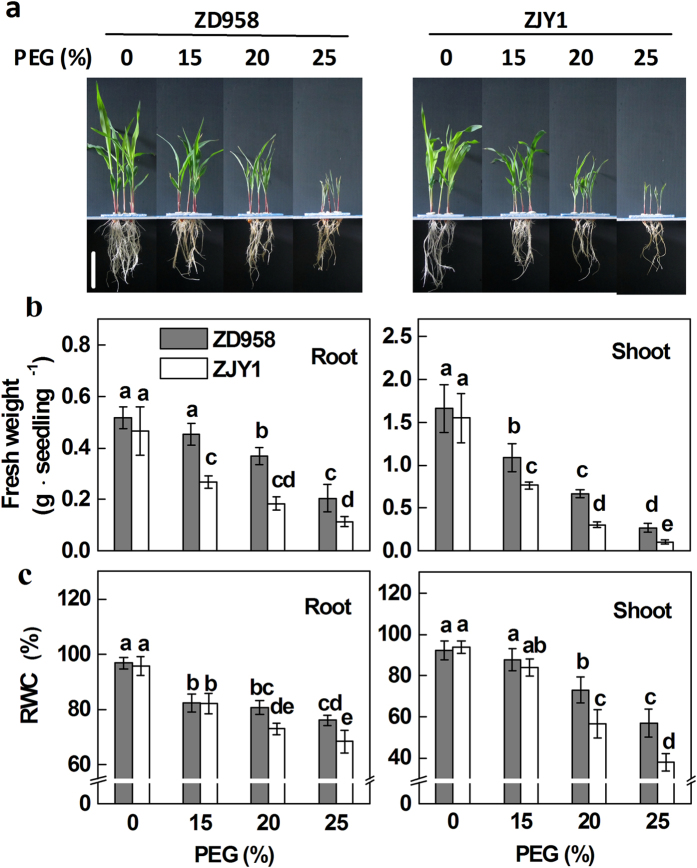
Phenotypic and physiological comparisons of two maize cultivars upon PEG stress. (**a**) 5-d-old seedlings of ZD958 and ZJY1 were transferred to half-strength Hoagland solutions containing the indicated concentrations of PEG-6000 (PEG) for another 5 d. Photographs were then taken. Bar = 10 cm. Meanwhile, fresh weight (**b**) and relative water content (RWC; **c**) were measured in both root and shoot tissues. Data are presented as means ± SE (5 root or shoot parts per experiment performed three times). Bars with different letters denote significant differences according to multiple comparisons (*P* < 0.05).

In this study, two highly productive maize cultivars, namely the osmotic-stress tolerant Zhengdan 958 (ZD958) and the sensitive Zhongjiangyu No.1 (ZJY1), were selected for osmotic stress sensitivity analysis. 5-d-old seedlings were treated with different concentrations of PEG-6000 for another 5 d, and phenotypic changes of both root and shoot tissues were monitored. PEG-6000 treatment significantly inhibited maize seedling growth in a dose-dependent manner (Fig. 1a), and the growth inhibition was more pronounced in ZJY1 than that of ZD958. For example, compared to the PEG-free control samples, the fresh weight of root tissues in ZD958 with 15%, 20%, and 25% PEG-6000 treatments decreased to 86.5 ± 7.7%, 70.9 ± 6.5%, and 39.6 ± 10.2%, and with 53.4 ± 5.4%, 39.4 ± 5.6%, and 24.5 ± 4.2% in ZJY1 (Fig. 1b). A similar trend was observed for the shoot tissues of these two maize cultivars under different concentrations of PEG-6000.

To further compare responses of ZD958 and ZJY1 under PEG-6000 treatment, relative water content (RWC) was analyzed (Fig. 1c). Although no significant difference was observed in root and shoot tissues upon 15% PEG-6000 treatment in two cultivars, ZD958 exhibited higher RWC than those of ZJY1 under more severe stress conditions. Combined with the changes in fresh weight, these results confirmed thatZD958 is more osmotic stress tolerant than ZJY1 under our experimental conditions. Therefore, ZD958 was regarded as an osmotic stress tolerant cultivar and ZJY1 as a sensitive cultivar in this study. Additionally, 20% PEG-6000, a moderate concentration, was used in the following experiments.

### Both methane and soluble sugar content were increased in response to PEG-induced osmotic stress

**Figure 2:**
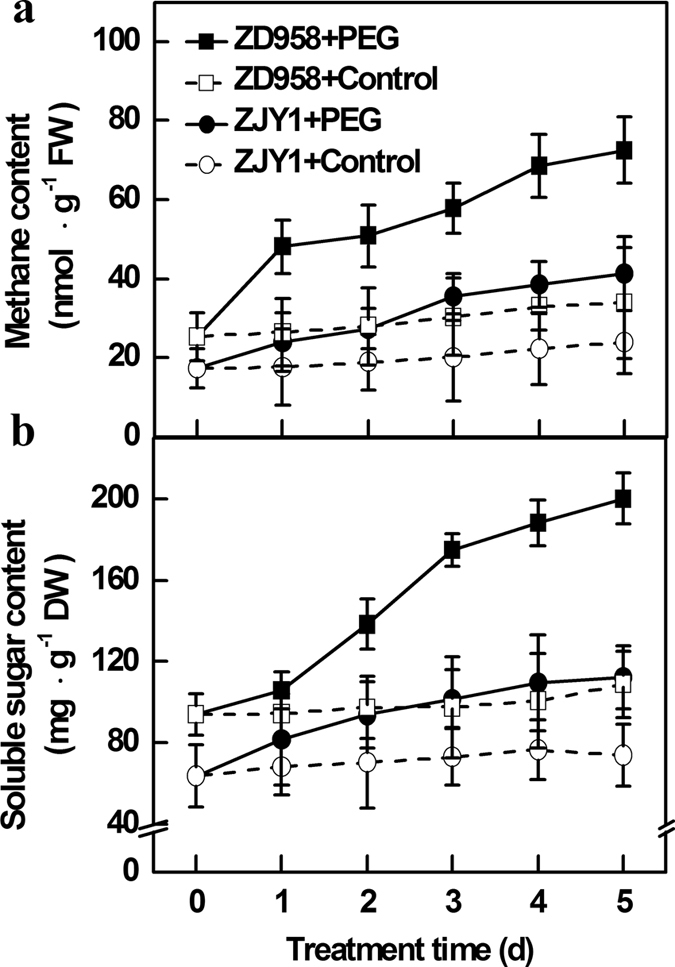
Time course analyses of methane production and soluble sugar content in two maize cultivars upon 20% PEG-6000 treatment. 5-d-old maize seedlings of ZD958 and ZJY1 were transferred to half-strength Hoagland solutions containing 20% PEG-6000 for another 5 d. Endogenous methane (**a**) and total soluble sugar (**b**) contents in root tissues were determined at the indicated time points. Control seedlings were incubated in Hoagland solution alone. Data are presented as means ± SE (5 root parts per experiment performed three times).

Previous results revealed that CH_4_ emission was increased under climatic stress conditions, and this phenomenon led to the “greenhouse effect”^4^,^16^. To investigate whether endogenous CH_4_ production was involved in PEG-induced osmotic stress, the endogenous CH_4_ production in root tissues of maize seedlings were monitored by gas chromatography (GC; Fig. 2a). Compared with the control conditions, CH_4_ production in root tissues of two cultivars continuously increased during a 5-d exposure to PEG-6000. Comparatively, CH_4_ production was more strongly enhanced in ZD958 (a tolerant cultivar) than in ZJY1 (a sensitive cultivar) under stress conditions. These results suggested that the inducement of CH_4_ production *in vivo* may be related to a protective response against osmotic stress. A similar increasing trend was observed in the soluble sugar content in the root tissues of ZD958 (in particular) and ZJY1 upon stress (Fig. 2b). Additionally, we observed that under control conditions, a higher CH_4_ or sugar content was detected in root tissues of ZD958 than that of ZJY1. Combined with the corresponding phenotypes (Fig. 1), these results clearly indicated a possible interrelationship between CH_4_ production and soluble sugar content in maize seedlings upon PEG-6000 treatment.

### PEG-induced osmotic stress was attenuated by CH_4_

Since sugar metabolism and signaling function exist as part of an integrated redox system, including quenching ROS and contributing to stress tolerance, especially in tissues or organelles with high soluble sugar concentrations^27^, we asked the question whether exposure to CH_4_ could reduce oxidative damage in maize plants upon PEG stress, so as to adapt to osmotic stress.

**Figure 3:**
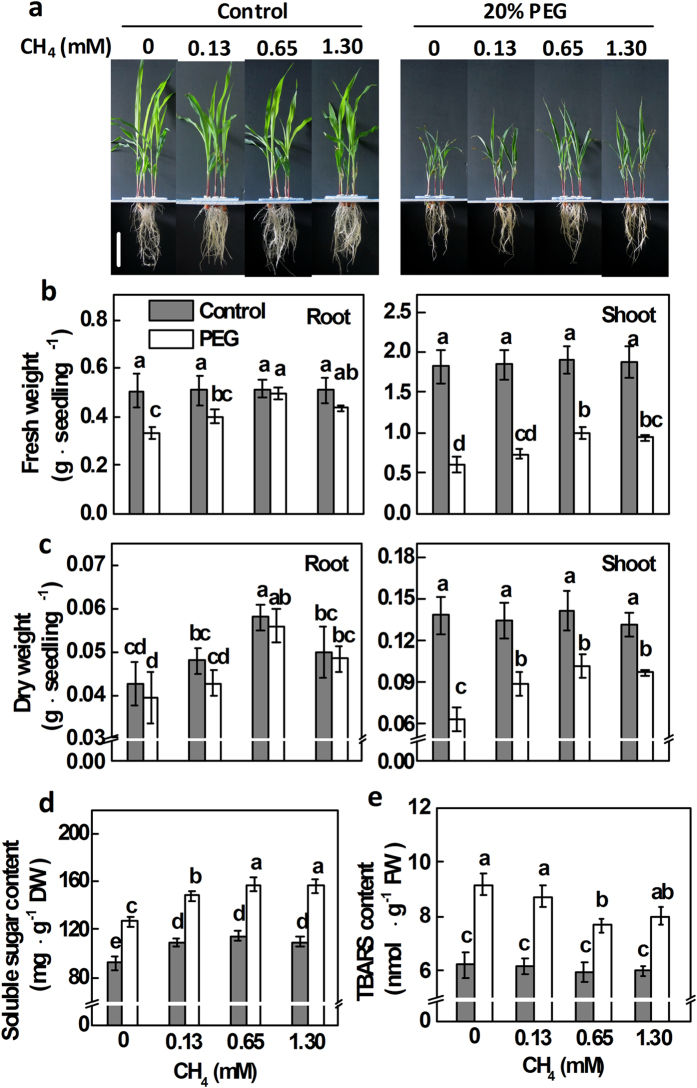
Effects of CH_4_ pretreatment on seedling growth, soluble sugar content, and lipid peroxidation in maize seedlings of ZD958 upon 20% PEG-6000 treatment. (**a**) 5-d-old seedlings were preincubated in the solution containing the indicated concentrations of CH_4_ for 1 d, and then transferred to half-strength Hoagland solutions with or without 20% PEG-6000 for another 5 d. Photographs were then taken. Bar = 10 cm. Meanwhile, fresh weight (**b**) and dry weight (**c**) were measured in both root and shoot tissues. The contents of soluble sugar (**d**) and TBARS (**e**) in root tissues were also determined. Control seedlings were incubated in Hoagland solution alone. Data are presented as means ± SE (5 root or shoot parts per experiment performed three times). Bars with different letters denote significant differences according to multiple comparisons (*P* < 0.05).

Different concentrations of CH_4_ (0.13, 0.65, and 1.30 mM CH_4_) were used to mimic endogenous CH_4_-related responses in ZD958 under PEG stress. Our experiments showed that pretreatments with CH_4_ partially alleviated the loss of fresh and dry weight in both root and shoot tissues of ZD958 caused by PEG (Fig. 3a–c). The maximal protective response was observed in 0.65 mM CH_4_-pretreated plants. When CH_4_ was applied alone, only dry weight in root tissues of ZD958 was obviously increased, by comparison with the control samples. Soluble sugar content was significantly enhanced by PEG-6000, which was further strengthened after CH_4_ pretreatment, with maximal responses at 0.65 and 1.30 mM (Fig. 3d). To further assess the protective effects of CH_4_, PEG-induced oxidative damage to cell membranes in root tissues was investigated. Because of the stressed plant tissues containing anthocyanin and other interfering compounds, the TBARS content, an important indicator of lipid peroxidation and free radical generation, were measured. Treatment with 20% PEG-6000 for 5 d caused a significant increase in TBARS content compared with the control samples (Fig. 3e). By contrast, the amount of the increased TBARS content triggered by PEG-6000 was reduced by CH_4_ pretreatment, with the maximal reduction observed at 0.65 mM CH_4_. No significant changes occurred in CH_4_-pretreated alone samples. Therefore, 0.65 mM CH_4_ was applied in the following experiments.

**Figure 4:**
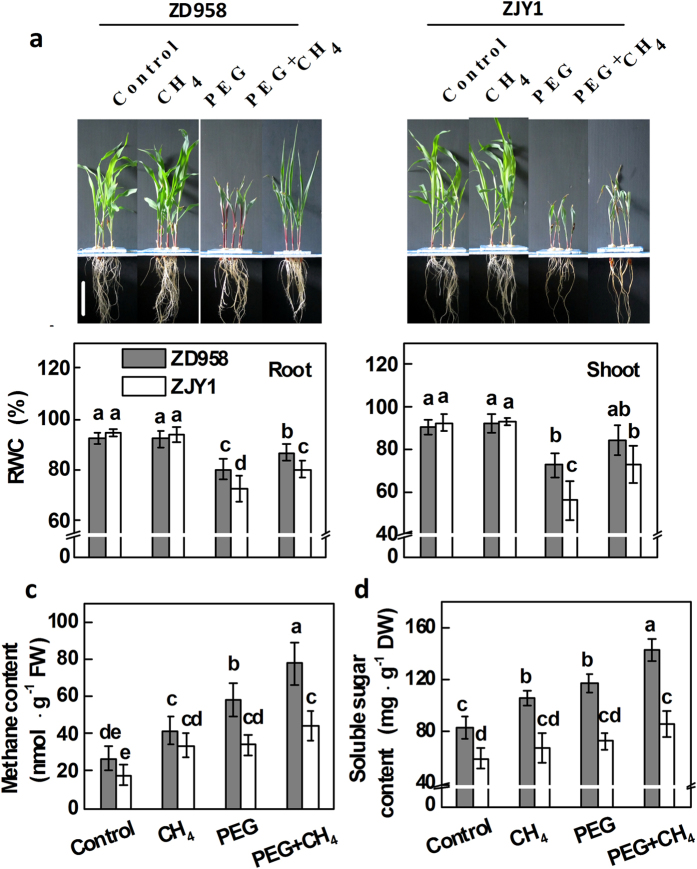
Comparison of different responses to CH_4_ pretreatment between ZD958 and ZJY1 upon 20% PEG-6000 treatment. (**a**) 5-d-old maize seedlings were preincubated in the solution containing 0.65 mM CH_4_ for 1 d, and then transferred to half-strength Hoagland solutions with or without 20% PEG-6000 for 5 d. Photographs were then taken. Bar = 10 cm. (**b**) Relative water content (RWC) was measured in both root and shoot tissues. Meanwhile, the contents of endogenous methane (**c**) and total soluble sugar (**d**) in root tissues were determined. Control seedlings were incubated in Hoagland solution alone. Data are presented as means ± SE (5 root or shoot parts per experiment performed three times). Bars with different letters denote significant differences according to multiple comparisons (*P* < 0.05).

To further investigate the regulatory roles of CH_4_ in PEG-induced osmotic stress, we compared the differences between ZD958 and ZJY1 after the pretreatment with CH_4_ (Fig. 4). CH_4_ alone could not influence the RWC in the root and shoot tissue of two cultivars. However, there were higher levels of RWC in both root and shoot tissues of ZD958 than those of ZJY1 upon CH_4_ followed by PEG-6000 stress (Fig. 4b). As expected, CH_4_ pretreatment increased endogenous CH_4_ and soluble sugar concentrations in PEG-treated plants, especially in root tissues of ZD958 than ZJY1 (Fig. 4c,d). Exogenously applied CH_4_ alone increased endogenous CH_4_ production to some extent. A similar induction was observed upon PEG-6000 treatment, particularly in ZD958. Therefore, we deduced that CH_4_ may protect against PEG-induced osmotic stress by improving sugar content.

### Expression of genes responsible for sugar metabolism

**Figure 5:**
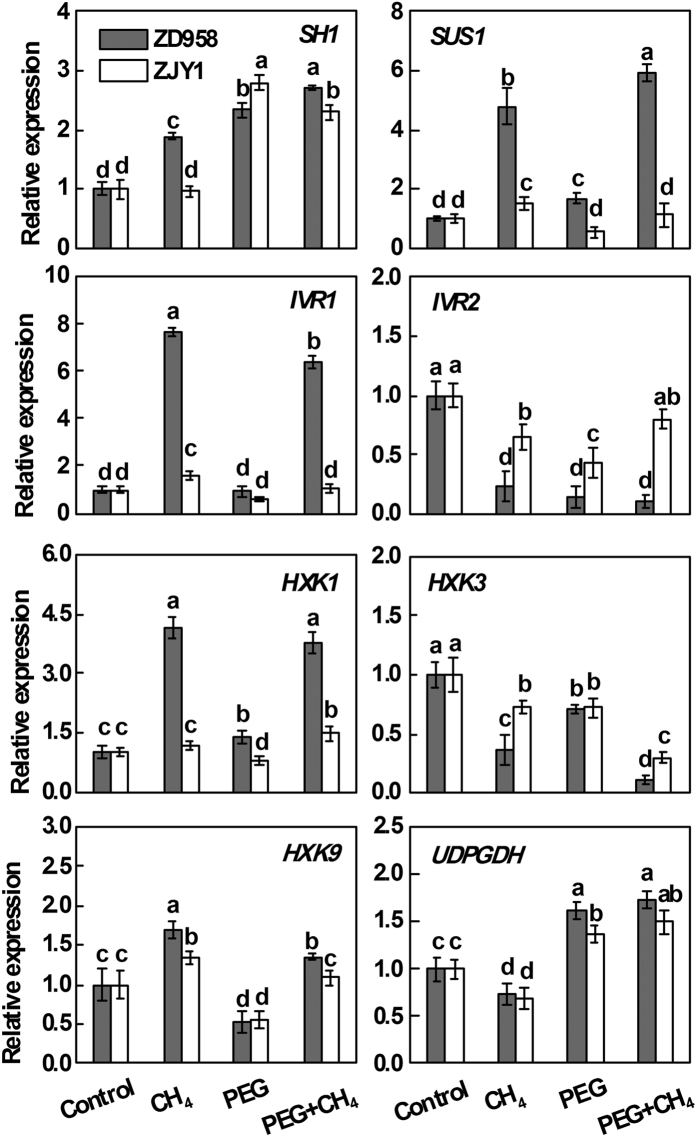
Regulation of transcripts related to sugar metabolism and signaling pathways by CH_4_. 5-d-old maize seedlings of ZD958 and ZJY1 were preincubated in the solution containing 0.65 mM CH_4_ for 1 d, and then transferred to half-strength Hoagland solutions with or without 20% PEG-6000 for 2 d. Relative gene expression of *sucrose synthase 1 (SH1*; X02400), *sucrose synthase 2 (SUS1*; L22296), *soluble acid invertase 1 (IVR1*; AF171874), *soluble acid invertase 2 (IVR2*; U31451), *hexokinase 1*/*3*/*9 (HXK1*/*3*/*9*; NM_001158821/XM_008676343/XM_008658658), *UDP*-*glucose dehydrogenase (UDPGDH*; EU961705) in root tissues were analyzed. Control seedlings were incubated in Hoagland solution alone. Data are presented as means ± SE (5 root parts per experiment performed three times). Bars with different letters denote significant differences according to multiple comparisons (*P* < 0.05).

To further establish the relationship between CH_4_ and sugar metabolism under PEG treatment, we analyzed the transcriptional profiles of some key genes in sugar metabolism and signaling pathways (Supplementary Fig. S1). Similar to the previous reports^27^,^28^, PEG treatment significantly increased the transcript levels of *sucrose synthase 1 (SH1*) and *UDP*-*glucose dehydrogenase (UDPGDH*), while decreased the gene expression of *soluble acid invertase 2 (IVR2*) and *hexokinase 3 (HXK3*) in root tissues of two maize cultivars (Fig. 5). Compared with PEG alone, the transcript levels of *sucrose synthase 2 (SUS1*), *soluble acid invertase 1 (IVR1*), and *hexokinase 1*/*9 (HXK1*/*9*), were dramatically enhanced by CH_4_ pretreatment in ZD958, a tolerant cultivar. However, no such striking differences were observed in root tissues of ZJY1 (a sensitive cultivar), showing no significant changes or weak induction in these three transcripts. These results indicated that CH_4_ conferred plant tolerance against osmotic stress by improving sugar metabolism.

### CH_4_ regulated redox status in response to PEG treatment

The experiments described above indicated that CH_4_ pretreatment was able to reduce TBARS content in PEG-stressed root tissues (Fig. 3e). Since PEG-induced oxidative stress in root tissues was associated with ROS production^29^, the antioxidant enzymes responsible for ROS scavenging were investigated. The total activities of SOD, POD, and CAT in root tissues of ZD958 were higher than those of ZJY1 under the control conditions (Supplementary Fig. S2). Although the SOD activities were approximately similar in the two maize cultivars under different treatments, the total activities of POD and CAT were increased by PEG treatment in both cultivars. The only significant effect of CH_4_ pretreatment was a decrease in POD activity in root tissues of ZD958.

**Figure 6:**
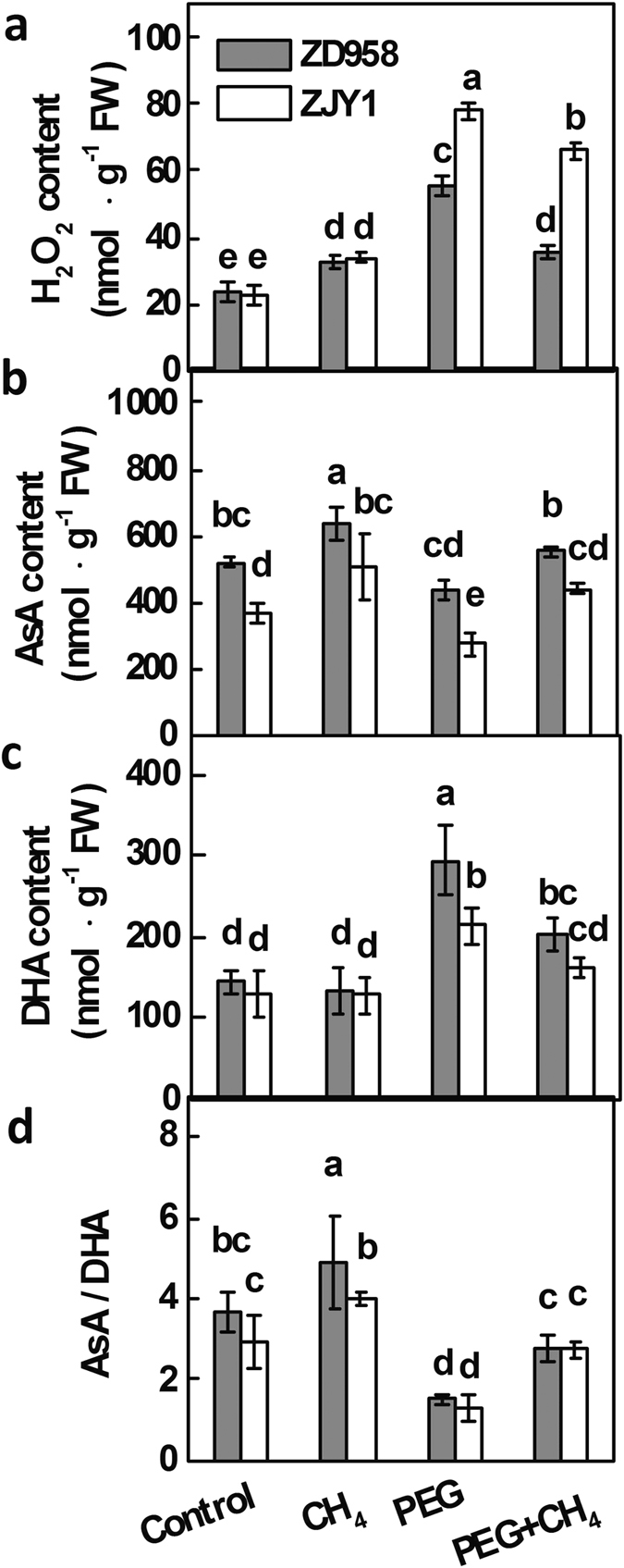
Regulation of redox state by CH_4_. 5-d-old maize seedlings of ZD958 and ZJY1 were preincubated in the solution containing 0.65 mM CH_4_ for 1 d, and then transferred to half-strength Hoagland solutions with or without 20% PEG-6000 for 2 d. Afterwards, the contents of endogenous H_2_O_2_ (**a**) in root tissues was analyzed. Meanwhile, the contents of reduced ascorbic acid (AsA; **b**), dehydroascorbate (DHA; **c**), and AsA/DHA ratio (**d**) in root tissues were determined according to the method described by Law *et al*.^51^. Control seedlings were incubated in Hoagland solution alone. Data are presented as means ± SE (5 root parts per experiment performed three times). Bars with different letters denote significant differences according to multiple comparisons (*P* < 0.05).

PEG stress-induced ROS has been demonstrated to cause oxidative damage to plants, and H_2_O_2_ and O_2_^−^ are believed to be the most important components. The effect of CH_4_ on the PEG-induced ROS overproduction was further investigated. Our results showed that PEG induced significant increases in the levels of H_2_O_2_ and O_2_^−^ in root tissues of both cultivars (Fig. 6a; Supplementary Fig. S3). CH_4_ pretreatment significantly reduced PEG-induced ROS production, with more effects observed in root tissues of ZD958 than in ZJY1.

Since the glutathione-ascorbate cycle is a metabolic pathway that detoxifies H_2_O_2_, AsA and GSH homeostasis were analyzed. CH_4_ alone enhanced the reduced AsA content and AsA/dehydroascorbate (DHA) ratio in both ZD958 and ZJY1 when compared to the control conditions (Fig. 6b–d). By contrast, PEG decreased AsA content and significantly increased DHA content, therefore resulting in a lower AsA/DHA ratio. However, CH_4_ pretreatment obviously blocked the decrease of AsA content and the increase of DHA level induced by PEG, thus resulting in the higher level of AsA/DHA ratio, in comparison with PEG alone. Moreover, higher reduced and total contents of ascorbate (AsA+DHA) were observed in ZD958 than ZJY1 under all of the tested conditions (Table 1).

As expected, treatment with PEG triggered an obvious increase in content of GSH and an decrease of GSSG in roots (Table 2). By contrast, CH_4_ pretreatment significantly eliminated the effects of PEG alone on GSH and GSSG contents. Meanwhile, a higher ratio of GSH/GSSG, an important parameter for the intracellular redox status, was observed in the CH_4_ pretreatment cultivars (ZD958 in particular) followed by PEG exposure, with respect to PEG alone samples. These results implied that CH_4_ may be involved in the reestablishment of redox status in PEG-treated maize seedlings via the modulation of AsA and GSH homeostasis.

### Expression of genes responsible for AsA metabolism

**Figure 7:**
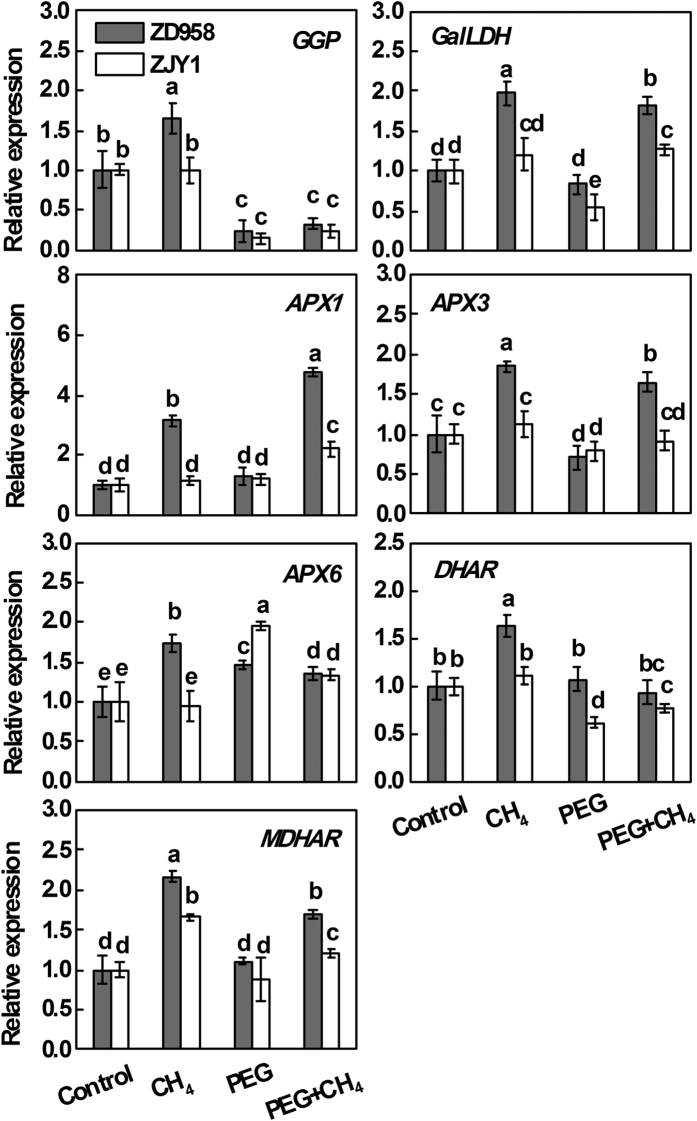
Regulation of transcripts related to ascorbic acid metabolism. 5-d-old maize seedlings of ZD958 and ZJY1 were preincubated in the solution containing 0.65 mM CH_4_ for 1 d, and then transferred to half-strength Hoagland solutions with or without 20% PEG-6000 for 2 d. Relative gene expression of *GDP*-*L*-*galactose phosphorylase (GGP*; DT943063), *L*-*galactono*-*1*,*4*-*lactone dehydrogenase (GalLDH*; DT943591), *ascorbate peroxidase 1 (APX1*; NM_001177011), *ascorbate peroxidase 3 (APX3*; NM_001159274), *ascorbate peroxidase 6 (APX6*; NM_001139033), *dehydroascorbate reductase (DHAR*; DR807318), and *monodehydroascorbate reductase (MDHAR*; CO461725) in root tissues were analyzed^55^. Control seedlings were incubated in Hoagland solution alone. Data are presented as means ± SE (5 root parts per experiment performed three times). Bars with different letters denote significant differences according to multiple comparisons (*P* < 0.05).

In order to investigate the relationship between CH_4_ and AsA metabolism, the transcriptional profiles of some key genes in AsA production and recycling (Supplementary Fig. S1) were analyzed. As shown in Fig. 7, CH_4_ pretreatment alone brought about much higher transcript levels of *GDP*-*L*-*galactose phosphorylase (GGP*), *L*-*galactono*-*1*,*4*-*lactone dehydrogenase (GalLDH*), *ascorbate peroxidase 1*/*3*/*6 (APX1*/*3*/*6*), *dehydroascorbate reductase (DHAR*), and *monodehydroascorbate reductase (MDHAR*) in ZD958; no such significant increases were observed in ZJY1 except for the gene expression of *MDHAR*. Compared with the control conditions, PEG treatment significantly decreased the transcript levels of *GGP* and *APX3* in both ZD958 and ZJY1, but increased the expression of *APX6*. Except for *APX1* and *APX6* in ZJY1, higher transcript levels of all the tested genes were observed in CH_4_ pretreatment alone than those in only PEG-6000 stress. After CH_4_ pretreatment followed by PEG stress, there were significant increases in the levels of *GalLDH, APX1*/*3*, and *MDHAR* mRNA in the root tissues of ZD958 (in particular) and ZJY1 compared to PEG alone, all of which were consistent with the decreased H_2_O_2_ concentration and high level of AsA/DHA ratio (Fig. 6a,d).

### Possible precursor(s) of CH_4_ production

Some compounds, such as amino acid _L_-methionine, have been suggested as likely sources of CH_4_ emission partly due to the degree of methylation^1^,^4^,^7^,^12^. To investigate whether these compounds could also induce endogenous CH_4_ production in maize, _L_-methionine contents was analyzed. In comparison with the control conditions, CH_4_ alone had no significant effect on _L_-methionine production. _L_-Methionine production in the two cultivars was significantly increased by PEG-6000 stress, especially in ZD958 (Supplementary Fig. S4). Pretreatment with _L_-methionine induced endogenous CH_4_ production, especially in ZD958. Together, although the other source(s) for endogenous CH_4_ production can’t be ruled out, our results clearly confirm that _L_-methionine is a possible precursor of endogenous CH_4_ production.

## Discussion

A comprehensive understanding of CH_4_ in plants is that the living plants and fungi can not only emit CH_4_ to the atmosphere^7^, but also firstly produce CH_4_ *in vivo*^8^. Similar phenomenon occurred in nitric oxide^9^ and carbon monoxide^10^, etc. Although CH_4_ emission and/or release have previously considered as a non-enzymatic process rather than an enzyme-mediated process^4^, whether or how plants produce endogenous CH_4_ production is still elusive. Here, we demonstrated that PEG treatment triggered endogenous CH_4_ production in maize cultivars with an approximately time-dependent manner (Fig. 2). Thus, our study suggested that CH_4_ production in plants can be induced by environmental stimuli, including osmotic stress and salinity^23^.

Among the potential chemical compounds related to CH_4_ emission and/or release, it was reported that methyl compounds can act as precursors for CH_4_ formation under aerobic conditions^14^. For example, Keppler *et al*.^4^ suggested the role of methyl groups on pectin as a source for CH_4_ formation from plant tissues. After the digestion of pectin with pectin methyl esterase (PME), Bruhn *et al*.^15^ further observed a decrease of CH_4_ efflux in solutions of *Citrus limon* fruit pectin after UV-B treatment, in comparison with stress alone. Partly consistent with above results, our tests showed that seedling roots of ZD958, an osmotic stress tolerant cultivar, contain a relatively higher level of pectin upon PEG stress, compared to that in the sensitive cultivar ZJY1 (Supplementary Fig. S5). Endogenous CH_4_ production was progressively triggered by PEG stress, particularly in ZD958 (Fig. 2). However, the causal link between pectin and CH_4_ production is not fully elucidated, and endogenous CH_4_ production might be mediated by multiple pathways^5^,^12^,^14^.

Subsequent results revealed that the endogenous _L_-methionine production was induced by PEG stress (Supplementary Fig. S4a). The exogenous addition of _L_-methionine increased endogenous CH_4_ levels both in ZD958 (in particular) and ZJY1 (Supplementary Fig. S4b). A similar result was found in the papers from Frank Keppler’s lab^7^,^30^. Based on the addition of ^13^CH_3_-Met and stable isotope measurements, the δ^13^C(CH_4_) values in the headspace of *L. angustifolia* and *P. sapidus* supplemented with ^13^C-methionine showed a continuous increase during the entire incubation period. Thus, _L_-methionine might be one of the precursors of CH_4_. Combined with our results in Supplementary Fig. S4, a stress-induced CH_4_ production from _L_-methionine might exist in plants^8^,^30^. Certainly, genetic and biochemical methods should be further provided to confirm this possibility.

Significant CH_4_ generation was previously demonstrated in animals^31^,^32^. It was further confirmed that CH_4_ is a critical molecule implicated in the anti-inflammatory diseases in mammals, and in protecting against ischemia reperfusion induced oxidative and nitrosative stresses^33^. This report triggers our interests to investigate the potential roles of CH_4_ in osmotic stressed plants.

Subsequent work showed that the increased endogenous CH_4_ production may benefit maize under osmotic stress. The following results support this conclusion: (i) the osmotic stress-tolerant maize cultivar ZD958 exhibited more CH_4_ production than the sensitive cultivar ZJY1 (Figs 1 and 2a); (ii) pretreatments with 0.65 mM CH_4_, which enhanced CH_4_ production in our experimental conditions (Fig. 4c), could significantly alleviate PEG-6000-induced symptoms of osmotic stress in both root and shoot tissues, including the improvement of seedling growth stunt (Fig. 3a–c); (iii) CH_4_ significantly blocked the increase in ROS production and TBARS content caused by PEG-6000 stress (Figs 3e and 6a, Supplementary Fig. S3); meanwhile, both AsA and GSH homeostasis were reestablished by CH_4_ (Tables 1 and 2, Fig. 6b–d). These finding parallels the situation encountered in animals, in which CH_4_ was shown to inhibit intestinal superoxide anion generation parallel to decreased activity of myeloperoxidase (MPO), a ROS producing enzyme^32^. Since the maintenance of ROS homeostasis has been linked to the increased tolerance of plants to a wide range of environmental stresses, our results preliminarily suggested that CH_4_ may enhance the adaptive plant responses against osmotic stress by alleviating PEG-induced oxidative damage.

A beneficial role of CH_4_ against salinity stress has been recently reported in alfalfa plants, which was mainly attributed to the induction of antioxidant defence^23^. To investigate the regulatory mechanism of CH_4_ on redox status, the total activities of SOD, POD, and CAT were determined in PEG-stressed plants (Supplementary Fig. S2). Unlike no obvious changes of SOD and CAT activities, the reduction of POD activity caused by CH_4_ only appeared in ZD958 upon PEG stress. In the presence of CH_4_ and PEG, the levels of H_2_O_2_ in two maize cultivars were lower than PEG alone (Fig. 6a), suggesting that CH_4_ might inhibit ROS production through a putative mechanism. Normally, plant PODs exist in a large number isozymatic forms and exhibit diverse functions depending on their substrates. Besides being responsible for scavenging H_2_O_2_, POD can generate H_2_O_2_ in the leaf apoplast of cowpea^34^. Therefore, we deduced that CH_4_-mediated reduction of POD might be partially responsible for the decreased H_2_O_2_ and TBARS contents (Figs 3e and 6a). Another possible explanation is that some potent non-enzymatic antioxidants, such as AsA, GSH, carotenoids, and tocopherols which directly interact with and detoxify ROS^25^, may be involved in the alleviation of PEG-induced oxidative damage triggered by CH_4_.

Sucrose is a pivotal integrating regulatory molecule that controls gene expression related to plant metabolism, stress resistance, growth and development^27^. The only known enzymatic pathways of sucrose cleavage in plants are catalyzed by invertase and sucrose synthases^35^. Similar to the responses of osmotic stress^28^, CH_4_ pretreatment followed by PEG stress differentially increased the transcript levels of *SH1, SUS1*, and *IVR1* in ZD958, the osmotic stress tolerant cultivar (Fig. 5), and may induce the production of glucose and UDP-glucose (Supplementary Fig. S1). Meanwhile, no significant changes or weak induction in these transcripts were observed in the osmotic stress sensitive cultivar. HXKs catalyze the conversion of glucose and fructose into hexose monophosphates, thereby permitting entry of carbon skeletons such as glucose-6-P into catabolism. Although it was reported that there are nine *HXKs* in maize, only *HXK1*/*3*/*9* contain stress-responsive *cis* elements in the promoter region^36^. In our experimental conditions, compared with PEG alone, the transcript levels of *HXK1*/*9* were dramatically enhanced by CH_4_ pretreatment followed by PEG in ZD958 (Fig. 5). Therefore, sugar metabolism or signaling may be involved in CH_4_-induced osmotic stress tolerance.

On the other hand, UDPGDH oxidizes UDP-D-glucose to UDP-D-glucuronate, which is a glycosyl donor for pectin biosynthesis^37^. Pectin content and its degradation affect cell wall integrity, which is closely linked with abiotic stress sensitivity^38^. It was reported that polysaccharide remodeling is an important process that controls the mechanical properties of the cell wall and the water status of plant cells under water deficit^39^. Here, we showed that CH_4_ pretreatment can slightly increase the pectin contents in ZD958 under PEG stress (Supplementary Fig. S5). As overexpression of genes related to the synthesis of matrix polysaccharides benefits the resistance of plants to water stress^40^, we therefore speculated that CH_4_-induced pectin production may be one of the mechanisms to increase osmotic stress tolerance in maize.

Interestingly, _D_-galacturonic acid derived from pectin breakdown could also be a source of AsA^41^. AsA is one of the key players in a redox hub that integrates metabolic information and environmental stimuli to different responses within the cellular signaling network^25^. A high level of endogenous AsA is essential to effectively maintain the antioxidant system that protects plants from oxidative damage^42^. In our experimental conditions, CH_4_ pretreatment could increase the reduced AsA contents and AsA/DHA ratio (Fig. 6b–d, Table 1), thus decreasing PEG-induced H_2_O_2_ production (Fig. 6a). The comparison of transcript profiles of some key enzymes in sugar and AsA metabolism (Figs 5 and 7), confirmed that CH_4_ may participate in sugar signaling, which in turn increases AsA production and recycling, resulting in the reduced oxidative damage caused by PEG. Finally, ROS homeostasis was reestablished, and osmotic stress tolerance was successfully enhanced.

Together, this is the first report of the physiological significance of endogenous CH_4_ production in the protection of higher plants from osmotic stress. This conclusion was confirmed by the improved biomass and RWC. Subsequent studies showed that at least in our experimental conditions, CH_4_ improves sugar and AsA metabolism, thus suppressing ROS production and resulting in the alleviation of PEG-induced osmotic stress in maize. Further genetic and molecular investigations are required for better understanding of the detailed molecular mechanisms of CH_4_-induced stress tolerance.

## Methods

### Preparation of culture solution containing methane and determination of methane content

The CH_4_ gas (99.9%, v/v) from a compressed gas cylinder (Nanjing Special Gas Co., China) was bubbled into 500 ml half-strength Hoagland solutions at a rate of 200 ml min^−1^ for at least 30 min. The corresponding methane-rich solution was then immediately diluted with half-strength Hoagland solutions to the concentrations required (0.13, 0.65, and 1.30 mM CH_4_), and maintained at a constant level for at least 12 h. Therefore, methane-rich solution was used twice a day. Similarly in animal research, CH_4_ was dissolved in 20 ml of physiological saline for 20 min at a speed of 200 ml min^−1^ to reach a supersaturated level^43^,^44^.

For analyzing endogenous CH_4_ production, seedling roots were homogenized with 5 ml sterile water and transferred to a vial. Sulphuric acid was added to digest plant material, which was adopted in the determination of carbon monoxide and hydrogen gas production^45^,^46^. The GC was calibrated using a secondary standard CH_4_ mixture (2.0 ppm CH_4_ in N_2_; Nanjing Special Gas Co., China). For the measurement of samples, 2 ml of the head space air in the vial was injected directly into carrier gas by syringe.

### Plant materials and growth conditions

Two maize (*Zea mays* L.) cultivars Zhengdan 958 (ZD958) and Zhongjiangyu No.1 (ZJY1) were used in this study. After soaking overnight, maize seeds were germinated on filter paper imbibed in distilled water at 25 °C in the darkness for 2 d. Uniform seedlings were then chosen and transferred to an incubator with a 14-h photoperiod at 25 ± 1 °C and 200 μmol m^−2^ s^−1^ irradiation. After growing for another 2 d, seedlings were incubated in half-strength Hoagland solutions with or without polyethylene glycol (PEG-6000) as described in the corresponding figure legends. After various treatments for 5 d or the indicated time points, plants were photographed, and shoot or root parts were sampled for used immediately or flash-frozen in liquid nitrogen, and stored at −80 °C for further analysis.

### Measurement of relative water content (RWC)

Relative water content (RWC) was measured following the method of Fukao *et al*.^47^. Fresh weight (FW) of root or shoot tissues were determined immediately after harvest, and then different tissues were floated on deionized water for 6 h. The rehydrated tissues were re-weighted to determine turgid weight (TW). Finally, different tissues were oven dried at 65 °C for 3 d, and dry weight (DW) of each sample was measured. RWC was calculated using the following equation: RWC (%) = ((FW - DW)/(TW - DW)) × 100%.

### Soluble sugar analysis

Total soluble sugar content was analyzed according to the spectroscopic method described by Yemm & Willis^48^. The anthrone reagent was prepared by dissolving 1.0 g anthrone in 50 ml ethyl acetate, and heated in a boiling water bath. Maize root tissues were crushed in 5 ml distilled water with a mortar and pestle. The suspensions were centrifuged at 10,000 *g* for 20 min, and the supernatants were collected. Total soluble sugars were analyzed by 100 μl of sample extract reacting with 0.5 ml anthrone reagent and 5 ml H_2_SO_4_. The mixture was incubated in a boiling water bath for 10 min. After cooling, the absorbance at 630 nm was determined. Glucose was used to prepare standard solutions for the calibration curve.

### Determination of thiobarbituric acid reactive substances (TBARS)

Lipid peroxidation was estimated by measuring the amount of TBARS as previously described by Hodges *et al*.^49^. Maize root tissues were homogenized with inert sand in 80:20 (v:v) ethanol:water, followed by centrifugation at 10,000 *g* for 10 min. Samples were crushed with either 20% trichloroacetic acid (TCA) and 0.01% butylated hydroxytoluene or 0.65% 2-thiobarbituric acid (TBA) solution. After heating in a boiling water bath for 30 min, the mixture was quickly cooled, and then centrifuged at 10,000 *g* for 10 min. The absorbance at 440, 532, and 600 nm was determined. The concentration of lipid peroxides was quantified in terms of TBARS amount using an extinction coefficient of 157 mM^−1^ cm^−1^ and expressed as nmol g^−1^ fresh weight (FW).

### Determination of H_2_O_2_, dehydroascorbate (DHA) and AsA contents

For the determination of endogenous H_2_O_2_ content^50^, maize root tissues (about 0.2 g) were ground with liquid nitrogen and homogenized with 0.2 M HClO_4_ at 4 °C. The suspensions were centrifuged at 10,000 *g* for 20 min, and the supernatants were collected. An aliquot of supernatant (500 μl) was added to 500 μl of assay reagent (0.5 mM ammonium ferrous sulfate, 50 mM H_2_SO_4_, 0.2 mM xylenol orange, and 200 mM sorbitol) and the absorbance at 560 nm was determined after 15 min of incubation at 30 °C. Standard curves were obtained by adding variable amounts of H_2_O_2_.

Reduced ascorbic acid (AsA) and dehydroascorbate (DHA) contents were measured according to the previous method^51^. After treatment, fresh root tissues were crushed and homogenized in cold 6% TCA immediately. AsA and DHA content was analyzed by 200 μl of sample extract reacting reagents. Afterwards, samples were vortex-mixed and incubated at room temperature for 1 min, and 0.1 ml of 10 mM dithiothreitol was added. Then 0.5 ml of 10% TCA, 0.4 of 44% ortho-phosphoric acid, 0.4 ml of 4% 2,2′-dipyridyl in 70% ethanol, and 0.2 ml of 3% (w/v) FeCl_3_, were added. The mixture was incubated at 37 °C for 30 min, and the absorbance at 525 nm was determined. Standard curves were obtained by adding variable amounts of AsA.

Alternatively, another method for the determination of ascrobate was carried out according to the method reported by Ueda *et al*.^52^. For the measurement of reduced AsA, the reaction mixture consisted of 10 μL of 0.01 U μL^−1^ AO, 10 μL of sample, and 80 μL of 0.1 M potassium phosphate buffer (pH 7.0). For the measurement of oxidized AsA, the reaction mixture consisted of 4 mM DTT, 80 μL of 0.1 M potassium phosphate buffer (pH 7.8), and 10 μL of extract. In blank wells, the AO solution or DTT was substituted by potassium phosphate buffer (pH 7.8). After shaking the plate for 5 s, continuous absorbance reading at 265 nm was started by using UV spectrophotometry (UV1102II, Tianmei, China). Finally, total AsA was calculated.

### Determination of GSH and GSSG contents

GSH and GSSG contents were assayed according to the procedure of Gronwald *et al*.^53^. GSH content in root of maize was analyzed by the 5,5′-dithio-bis-(2-nitrobenzoic acid) (DTNB)-glutathione reductase (GR) recycling assay. GSSG was determined by the same method in the presence of 2-vinylpyridine. Absorbance at 412 nm was determined, and the GSH/GSSG ratio was calculated.

### RNA isolation and real-time RT-PCR analysis

Total RNA was isolated from maize root tissues with Trizol reagent (Invitrogen, Carlsbad, CA USA) according to the manufacturer’s instructions. Real-time RT-PCR experiments were performed using a Mastercycler^®^ ep *realplex* real-time PCR system (Eppendorf, Germany) with SYBR pre-mixture kit (BioTeke, China). Using specific primers (Supplementary Table S1), the expression levels of corresponding genes were normalized against two internal control genes *β*-*tubulin (TUB*) and *actin1 (ACT1*) under identical conditions^28^. The efficiency and specificity of all the primers were checked by both melting curve analysis and agarose gel in our experimental conditions. The data were based on three independent biological replicates and each sample was prepared in triplicate.

### Antioxidant enzyme activity assay

As previously described by Huang *et al*.^54^, superoxide dismutase (SOD, EC 1.15.1.1) activity was assayed by measuring its capacity of inhibiting the photochemical reduction of NBT. One unit of SOD (U) was defined as the amount of crude enzyme extract required to inhibit the reduction rate of NBT by 50%. Guaiacol peroxidase (POD, EC 1.11.1.7) activity was determined by measuring the oxidation of guaiacol (extinction coefficient 26.6 mM^−1^ cm^−1^) at 470 nm. Catalase (CAT, EC 1.11.1.6) activity was measured by monitoring the consumption of H_2_O_2_ (extinction coefficient 39.4 mM^−1^ cm^−1^) at 240 nm. Protein was determined by the method of Bradford^52^, using bovine serum albumin (BSA) as the standard.

### Statistical analysis

All data presented were the mean values of three independent experiments. Each value was expressed as means ± SE. Statistical analysis was performed using SPSS 16.0 software. Differences among treatments were analyzed by one-way ANOVA taking *P* < 0.05 as significant according to multiple comparisons.

## Additional Information

**How to cite this article:** Han, B. *et al*. Methane protects against polyethylene glycol-induced osmotic stress in maize by improving sugar and ascorbic acid metabolism. *Sci. Rep.* **7**, 46185; doi: 10.1038/srep46185 (2017).

**Publisher's note:** Springer Nature remains neutral with regard to jurisdictional claims in published maps and institutional affiliations.

## Supplementary Material

Supplementary Information

## Figures and Tables

**Table 1:** Reduced and total AsA concentration.

Treatment	Reduced AsA (nmol·g^−1^ FW)		Total AsA (nmol·g^−1^ FW)	
	ZD958	ZJY1	ZD958	ZJY1
Control	485.8 ± 32.6B	364.3 ± 39.4DE	563.7 ± 45.3bc	449.5 ± 41.4d
CH_4_	554.3 ± 27.5A	493.2 ± 45.1B	682.2 ± 29.2a	577.3 ± 30.8b
PEG	432.4 ± 37.9C	324.4 ± 29.8E	689.6 ± 30.3a	443.2 ± 32.1d
PEG + CH_4_	498.4 ± 46.3B	389.8 ± 37.7D	702.1 ± 38.9a	540.8 ± 43.4c

5-d-old maize seedlings of ZD958 and ZJY1 were preincubated in the solution containing 0.65 mM CH_4_ for 1 d, and then transferred to half-strength Hoagland solutions with or without 20% PEG-6000 for 2 d. Afterwards, the contents of reduced and total ascorbic acid (AsA) in root tissues were determined by using ascorbate oxidase (AO) method reported by Ueda *et al*.^52^. Control seedlings were incubated in Hoagland solution alone. Data are presented as means ± SE (5 root parts per experiment performed three times). Within each set of experiments, bars with different letters denote significant differences according to multiple comparisons (*P* < 0.05).

**Table 2:** Changes in GSH homeostasis.

Treatment	GSH (nmol·g^−1^ FW)		GSSG (nmol·g^−1^ FW)		GSH/GSSG	
	ZD958	ZJY1	ZD958	ZJY1	ZD958	ZJY1
Control	39.3 ± 2.6D	28.3 ± 2.4D	56.7 ± 3.3b	49.5 ± 3.4c	0.69	0.57
CH_4_	37.6 ± 2.5D	24.2 ± 2.1D	68.2 ± 4.2a	57.3 ± 3.8b	0.55	0.42
PEG	103.4 ± 5.9A	74.4 ± 3.8C	48.6 ± 3.3d	43.2 ± 3.1d	2.12	1.72
PEG + CH_4_	98.7 ± 4.3B	69.8 ± 3.7C	52.1 ± 3.9c	49.4 ± 3.1c	1.89	1.41

5-d-old maize seedlings of ZD958 and ZJY1 were pre-incubated in the solution containing 0.65 mM CH_4_ for 1 d, and then transferred to half-strength Hoagland solutions with or without 20% PEG-6000 for 2 d. Afterwards, GSH and GSSG contents in the seedling roots were determined. Control seedlings were incubated in Hoagland solution alone. Data are presented as means ± SE (5 root parts per experiment performed three times). Within each set of experiments, bars with different letters denote significant differences according to multiple comparisons (*P* < 0.05).
