# Supplementary material for: Methane protects against polyethylene glycol-induced osmotic stress in maize by improving sugar and ascorbic acid metabolism
Source: Sci Rep. 2017 Apr 7;7:46185. doi: 10.1038/srep46185 (PMC5384014; doi:10.1038/srep46185)
Supplement: Supplementary Information [file srep46185-s1.doc]

**Research paper**

**Title:**

**Methane protects against polyethylene glycol-induced osmotic stress in maize by improving sugar and ascorbic acid metabolism**

**Bin Han^1#+^, Xingliang Duan^1+^, Yu Wang^1^, Kaikai Zhu^1^, Jing Zhang^1^, Ren Wang^2^, Huali Hu^3^, Fang Qi^1^, Jincheng Pan^1^, Yuanxin Yan^4^ & Wenbiao Shen^1*^**

^1^College of Life Sciences, Laboratory Center of Life Sciences, Nanjing Agricultural University, Nanjing 210095, China

^2^Institute of Botany, Jiangsu Province and Chinese Academy of Sciences, Nanjing 210014, China

^3^Institute of Agricultural Products Processing, Jiangsu Academy of Agricultural Sciences, Nanjing 210014, China

^4^ College of Agronomy, Nanjing Agricultural University, Nanjing 210095, China

**^*^**Corresponding. E-mail: [wbshenh@njau.edu.cn](mailto:wbshenh@njau.edu.cn)

^+^These authors contributed equally to this work.

Mail address: College of Life Science, Laboratory Center of Life Science, Nanjing Agricultural University, Nanjing 210095, China

**^#^** Present address: Hangzhou Dalton BioSciences, Ltd., Hangzhou 310053, China

Bin Han: [445032054@qq.com; Xingliang](mailto:445032054@qq.com; Xingliang) Duan: [2016216026@njau.edu.cn](mailto:2016216026@njau.edu.cn); Yu Wang: [1825602125@qq.com](mailto:1825602125@qq.com); Kaikai Zhu: [335190516@qq.com](mailto:335190516@qq.com); Jing Zhang: [1530087329@qq.com](mailto:1530087329@qq.com); Ren Wang: [wangren@126.com](mailto:wangren@126.com); Huali Hu: [59859583@qq.com](mailto:59859583@qq.com); Fang Qi: [1298270307@qq.com](mailto:1298270307@qq.com); Jincheng Pan: [2015116110@njau.edu.cn](mailto:2015116110@njau.edu.cn); Yuanxin Yan: [yuanxin.yan@njau.edu.cn](mailto:yuanxin.yan@njau.edu.cn); Wenbiao Shen: [wbshenh@njau.edu.cn](mailto:wbshenh@njau.edu.cn)

**Methods**

**Histochemical assays**

H_2_O_2_ and O_2_^-^ levels *in vivo* were determined by 3,3-diaminobenzidine (DAB) and nitroblue tetrazolium (NBT) staining, respectively^1^. Roots were immersed in freshly prepared DAB solution (pH 3.8), and then incubated in darkness at 22℃ for 1 h. Alternatively, seedlings were immersed in NBT solution in 10 mM potassium phosphate buffer (pH 7.8) containing 10 mM NaN_3_, and then incubated in darkness at 22℃ for 1 h. After incubation, the stained roots were placed in a solution containing acetic acid:glycerol:ethanol (1:1:3, v/v/v) at 95℃ for 10 min, and then stored in 95% ethanol until photographed (model Stemi 2000-C; Carl Zeiss, Germany).

**Free _L_-methionine analysis by mass spectrometry**

Protein extraction was performed by using Plant EXTRACTION KIT (Sigma), and the protein content was determined by the Bradford^2^. The protein liquid was digested with trypsin (Promega), desalted with C18 column (Empore) and freeze-dried. Finally, samples were then injected into a liquid chromatography (DIONEX Thermo Scientific), and free _L_-methionine contents were analyzed by tandem mass spectrometry using LTQ-Orbitrap mass spectrometer (Thermo Scientific).

**Measurement of pectin content**

Pectin content was measured following the previous method^3,4^. Root samples were thoroughly homogenized in 2 ml 95% ethanol using a mortar and pestle. Pectin material was prepared as the alcohol-insoluble residue after repeated washing with ethanol. The sample was centrifuged at 10000 *g* for 10 min and the supernatant was discarded. According to Ahmed & Labavitch^5^, the remaining material was hydrolyzed using 0.1 M NaOH. Pectin content was analyzed by 1.0 ml of sample extract reacting with 0.1 ml 0.15% carbazole-ethanol indicator and 5 ml H_2_SO_4_. The mixture was incubated in a boiling water bath for 15 min. After cooling, the absorbance at 530 nm was determined. Galacturonic acid was used as a calibration standard, and the root pectin content was expressed as galacturonic acid equivalents (GaE) per g DW.

**Fig. S1.**

**Sucrose**

**Glucose**

**Fructose**

**IVR**

**SUS (SH)**

**HXK**

**Glucose-6-P**

**UDP-Glucose**

**Fructose-6-P**

**UDP-Glucuronosyl**

**UDPGDH**

**Pectin**

**ROS**

**Methane**

**Glycolytic**

**pathway**

**GDP-L-Galactose**

**GGP**

**L-Galactose-1-P**

**L-Galactono-1,4-Lactone**

**GalLDH**

**Ascorbic acid**

**?**

**H_2_O_2_**

**MDHA**

**H_2_O**

**APX**

**DHA**

**MDHAR**

**GSH**

**GSSG**

**DHAR**

**PME**

**Polygalacturonic acid**

**Fig. S1.** The network of proposed sucrose and ascorbic acid metabolism pathways in plants^37,41^. The transcripts of some key enzymes indicated with light gray background were analyzed in this study. The dashed lines denote incompletely characterized pathways.

**Fig. S2.**

**Fig. S2.** Regulation of antioxidant enzyme activities by CH_4_. 5-d-old maize seedlings of ZD958 and ZJY1 were preincubated in the solution containing 0.65 mM CH_4_ for 1 d, and then transferred to half-strength Hoagland solutions with or without 20% PEG-6000 for 2 d. Afterwards, the total activities of superoxide dismutase (SOD; a), guaiacol peroxidase (POD; b), and catalase (CAT; c) in root tissues were analyzed. Control seedlings were incubated in Hoagland solution alone. Data are presented as means ± SE (5 root parts per experiment performed three times). Bars with different letters denote significant differences according to multiple comparisons (*P* < 0.05).

**b**

**c**

**a**

**Fig. S3**

**Fig. S3.** Redox homeostasis was reestablished by methane. 5-d-old maize seedlings of ZD958 and ZJY1 were preincubated in the solution containing 0.65 mM CH_4_ for 1 d, and then transferred to half-strength Hoagland solutions with or without 20% PEG-6000 for 2 d. Seedlings were collected and stained with 3,3-diaminobenzidine (DAB) or nitroblue tetrazolium (NBT) to visualize H_2_O_2_ or O_2_^-^ distribution, respectively. Control seedlings were incubated in Hoagland solution alone. Bar = 20 mm.


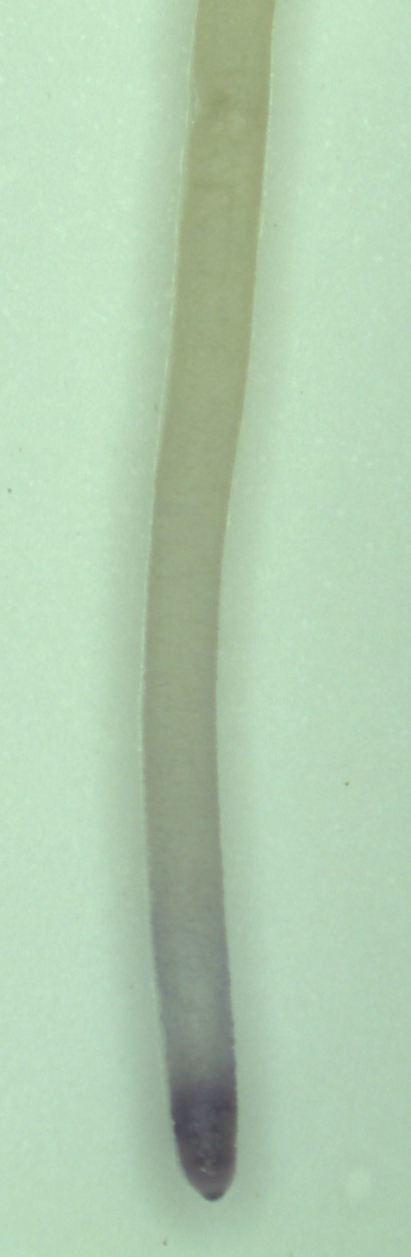

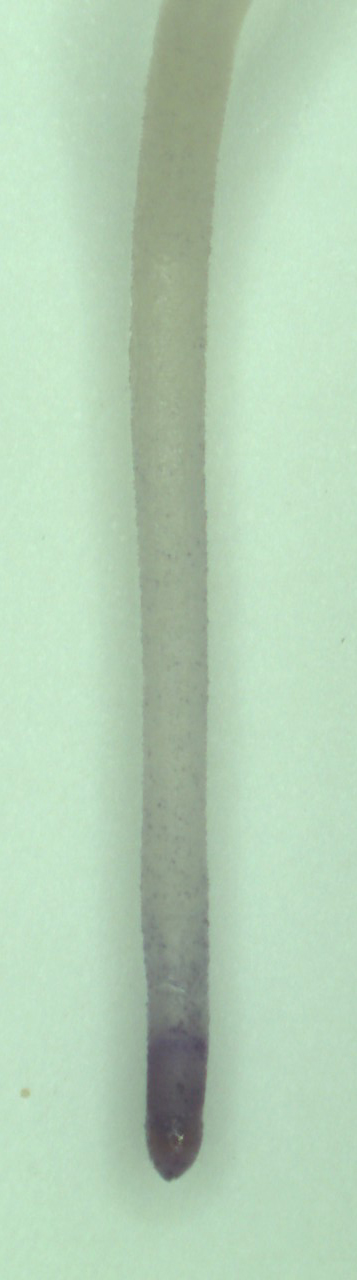

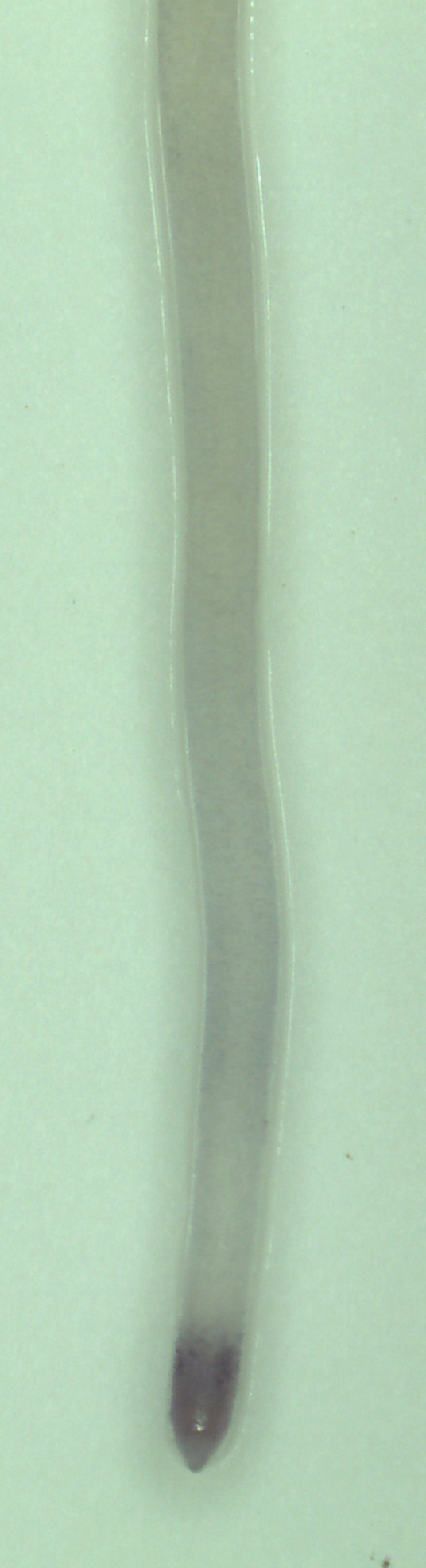

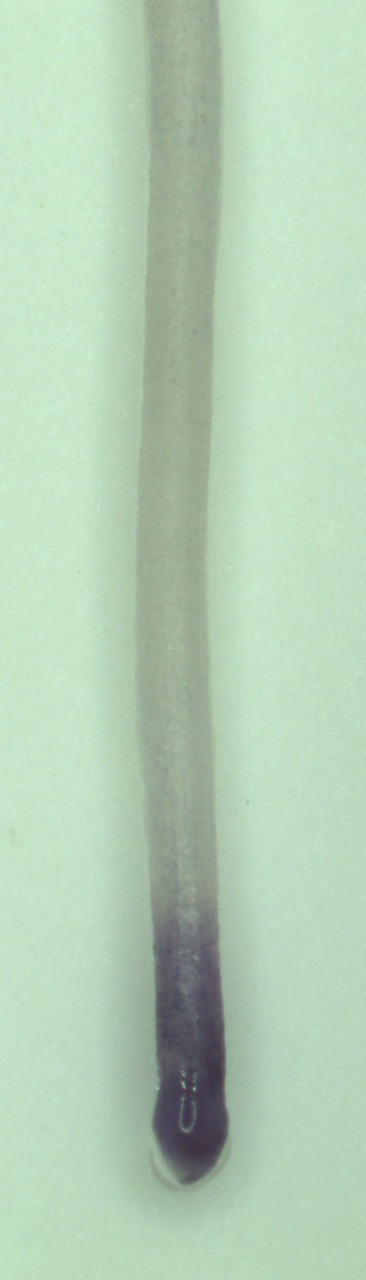

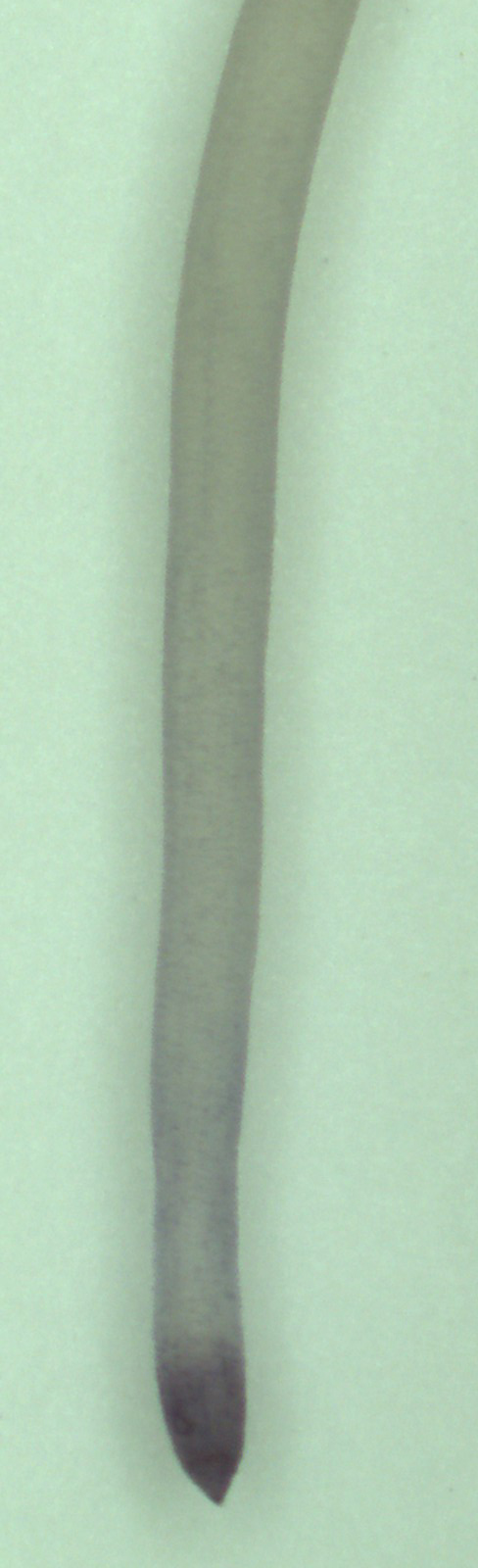

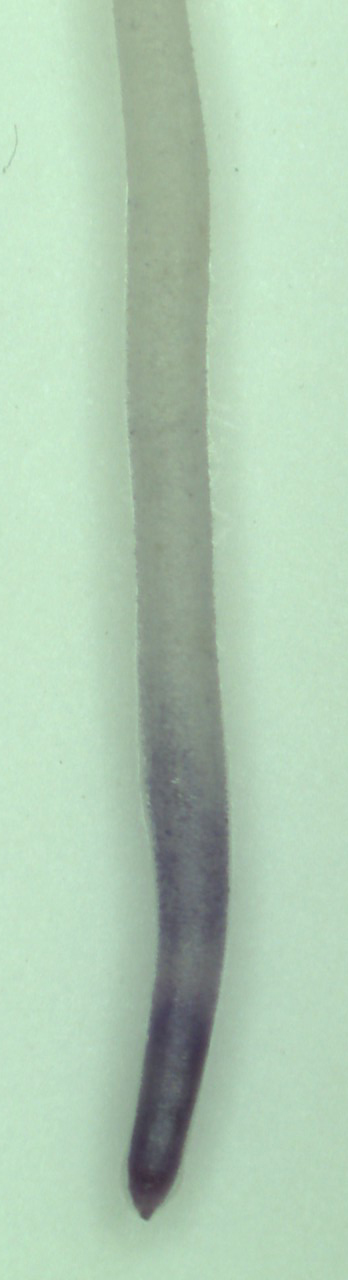

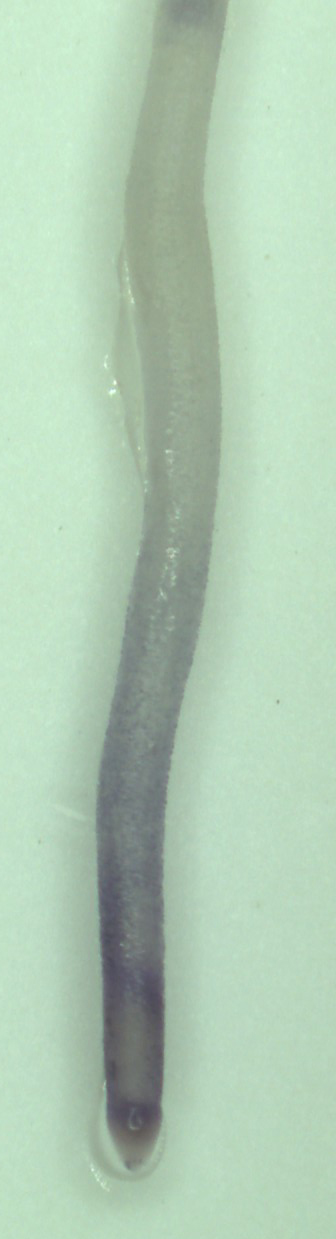

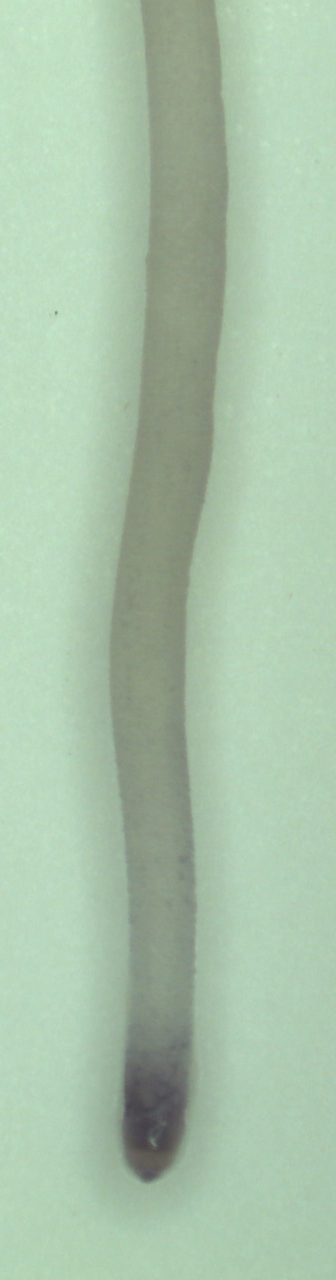

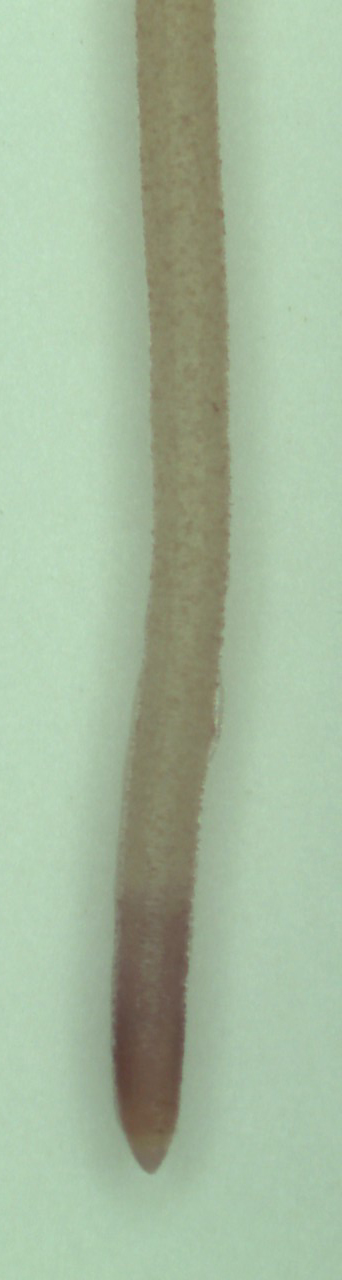

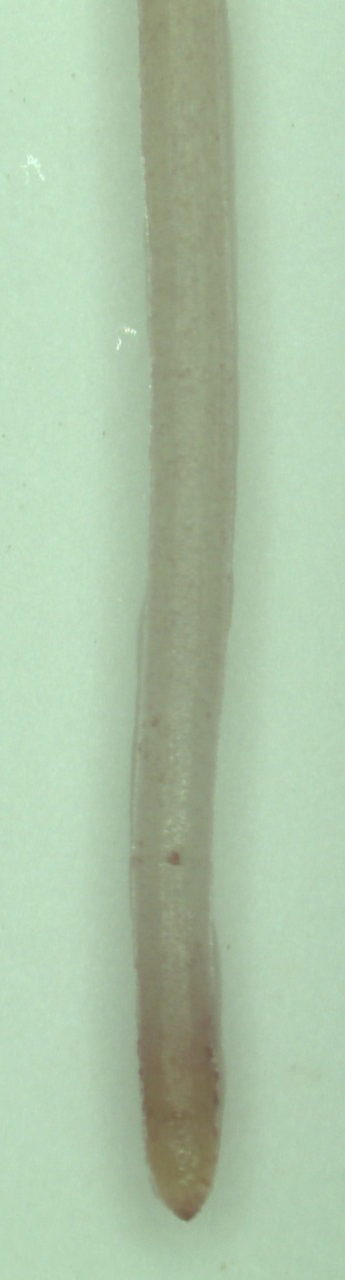

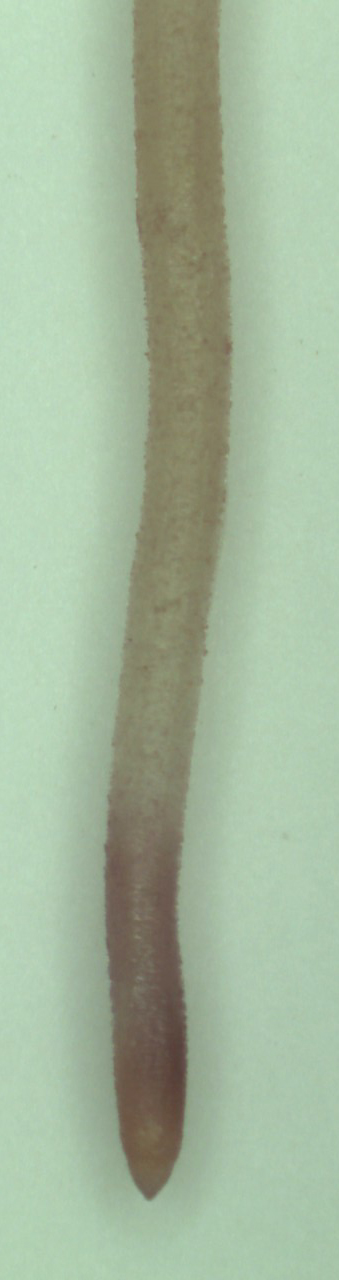

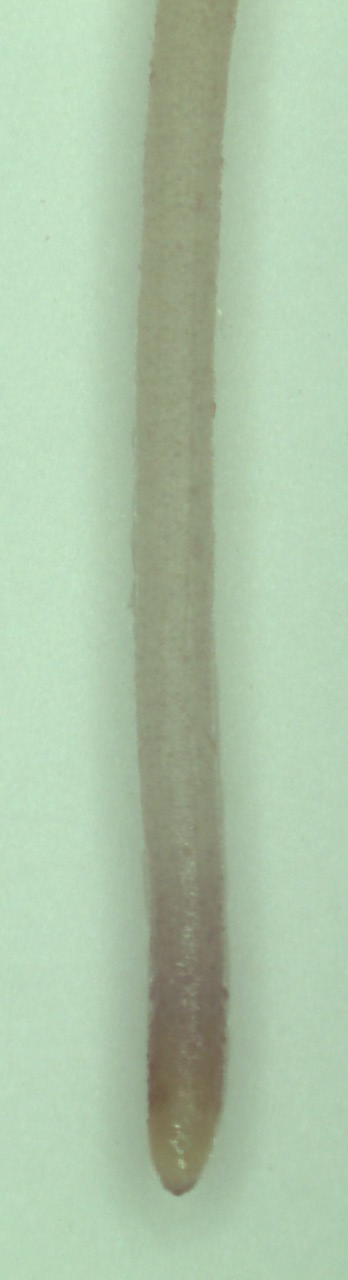


**DAB staining**

**NBT staining**


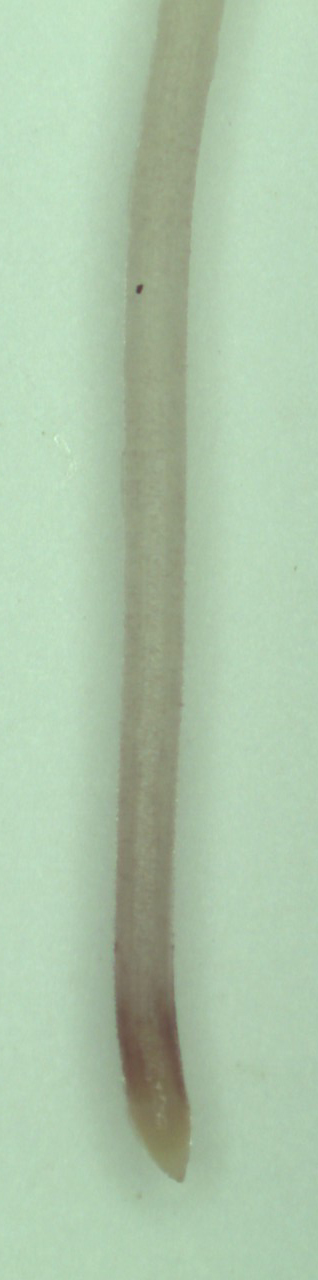

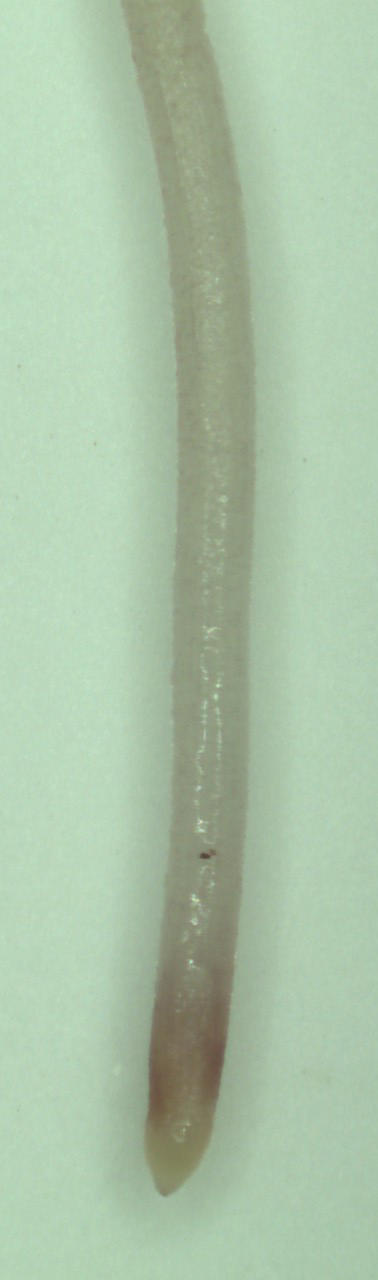

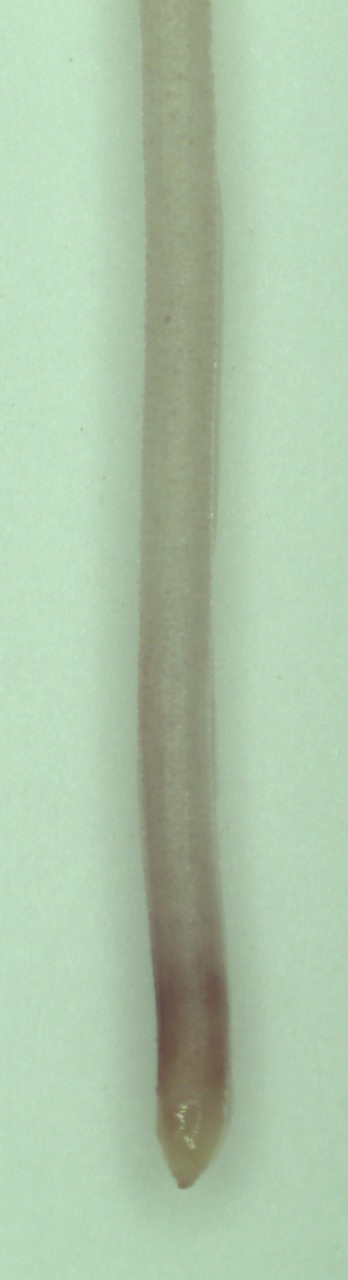

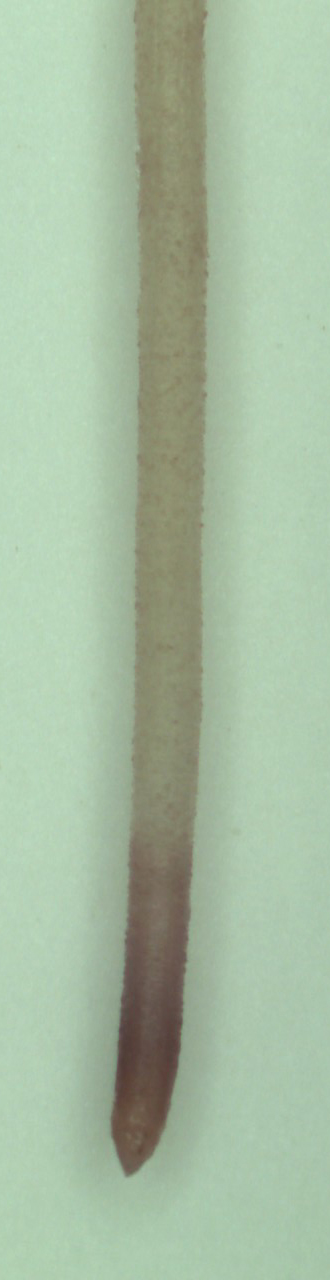


**ZD958**

**ZJY1**


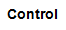

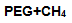

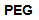

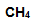

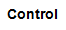

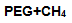

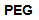

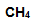


**Fig. S4.**

**Fig. S4.** A possible precursor of CH_4_ production. (a) 5-d-old maize seedlings of ZD958 and ZJY1 were preincubated in the solution containing 0.65 mM CH_4_ for 1 d, and then transferred to half-strength Hoagland solutions with or without 20% PEG-6000 for another 5 d. The _L_-methionine (Met) content was then measured in root tissues. (b) 5-d-old seedlings were treated with or without 1.5 mM Met for 6 h. Afterwards, the content of endogenous methane in root tissues was determined. Control seedlings were incubated in Hoagland solution alone. Data are presented as means ± SE (5 root parts per experiment performed three times). Bars with different letters denote significant differences according to multiple comparisons (*P* < 0.05).

**a**

**b**

**Fig. S5.**

**Fig. S5.** Comparison of pectin contents. 5-d-old maize seedlings of ZD958 and ZJY1 were preincubated in the solution containing 0.65 mM CH_4_ for 1 d, and then transferred to half-strength Hoagland solutions with or without 20% PEG-6000 for 2 d. Pectin content was measured and expressed as galacturonic acid equivalents (GaE) in root tissues. Control seedlings were incubated in Hoagland solution alone. Data are presented as means ± SE (5 root parts per experiment performed three times). Mean values with different letters denote significant differences according to multiple comparisons (*P* < 0.05).

**Table S1.** Primers used for real-time RT-PCR analysis.

| Accession number | Primer name | Sequences (5’→3’) |
| --- | --- | --- |
| X02400 | *SH1-F* | CACGACGATGATGTTGAATGAC |
|  | *SH1-R* | CGAGAAGCAAGTGGAGTGTGTC |
| L22296 | *SUS1-F* | ATGACGTGGCGCATGAGAT |
|  | *SUS1-R* | CCAGTAGAGGTCGGAGTTAGG |
| AF171874 | *IVR1-F* | CGGACGGCAGCCTCCAAACT |
|  | *IVR1-R* | CCATCTAGCACAGGGACCAAGC |
| U31451 | *IVR2-F* | ACGACCGCCACGACTACTAC |
|  | *IVR2*-R | GCCCATCCCTTGGACAC |
| NM_001158821 | *HXK1*-F | CCTGATTGCAGCTTCACAGT |
|  | *HXK1*-R | GTCATCTGCACGCTTGTCT |
| XM_008676343 | *HXK3*-F | GGATGTTGTTCCTCCGAAAC |
|  | *HXK3*-R | TCATGTGAGGTGTCATGGTG |
| XM_008658658 | *HXK9*-F | TTGGTGACGCTGCTGATAAT |
|  | *HXK9*-R | TCTTCCTGACTTCGCTCAGA |
| EU961705 | *UDPGDH*-F | AACGCCATCTCCGCCCTCT |
|  | *UDPGDH*-R | ACCCACCGAACCCAACACT |
| DT943063 | *GGP-F* | TTTTCCTGTTCCCCCAGTG |
|  | *GGP-R* | TAGTCCATCCTCCGTTTCAGC |
| DT943591 | *GalLDH-F* | CCAAGAAGAAGACCGTCACG |
|  | *GalLDH-R* | ATGTTGCACCAGTGCCATG |
| NM_001177011 | *APX1*-F | GCTTTTGTCGGGAGAGAAGG |
|  | *APX1*-R | GCTTCGGTGTAGTCAGCAAA |

| Accession number | Primer name | Sequences (5’→3’) |
| --- | --- | --- |
| NM_001159274 | *APX3*-F | GTGCCTGGACTACGAACCCTT |
|  | *APX3*-R | CACAAGAGGGCGGAAGACAG |
| NM_001139033 | *APX6*-F | CTCCCAACTGACAAGGCACTG |
|  | *APX6*-R | TACCCCAAAGGCACTCTGTG |
| DR807318 | *DHAR*-F | TGTCAGCGACTGATCTTAGCC |
|  | *DHAR*-R | TCACGGCTGAAAAGAGCCTT |
| CO461725 | *MDHAR*-F | TGGTGTTTCCTGAACCTTGG |
|  | *MDHAR*-R | ATGGCATCAGCATCAAACCAC |
| NM_001138798 | *TUB*-F | TCCTGGACAACGAGGCTATCTAT |
|  | *TUB*-R | TGTGAGATCAGCCTGTTCAAGTT |
| NM_001155179 | *ACT1*-F | TTGGGTCAGAAAGGTTCAGG |
|  | *ACT1*-R | GCACTTCATGTGGACAATGC |

**References**

1. Lv, W. T., Lin, B., Zhang, M. & Hua, X. J. Proline accumulation is inhibitory to Arabidopsis seedlings during heat stress. *Plant Physiol* **156**, 1921–1933 (2011).
2. Bradford, M. M. A rapid and sensitive method for the quantitation of microgram quantities of protein utilizing the principle of protein-dye binding. *Anal Biochem* **72,** 248–254 (1976).
3. Yang, J. L., Zhu, X. F., Zheng, C., Zhang, Y. J. & Zheng, S. J. Genotypic differences in Al resistance and the role of cell-wall pectin in Al exclusion from the root apex in Fagopyrum tataricum. *Ann Bot* **107,** 371–378 (2011).
4. Hu, H., Shen, W. & Li, P. Effects of hydrogen sulphide on quality and antioxidant capacity of mulberry fruit. *Int Food Sci Tech* **49,** 399–409 (2014).
5. Ahmed, A. E. R. & Labavitch, J. M. A simplified method for accurate determination of cell wall uronide content. *J Food Biochem* **1,** 361–365 (1978).
